# “Moving forward: a cross sectional baseline study of staff and student attitudes towards a totally smoke free university campus”

**DOI:** 10.1186/1471-2458-13-738

**Published:** 2013-08-08

**Authors:** Sharyn Burns, Jonine Jancey, Nicole Bowser, Jude Comfort, Gemma Crawford, Jonathan Hallett, Bree Shields, Linda Portsmouth

**Affiliations:** 1 Western Australian Centre for Health Promotion Research, School of Public Health, Curtin University, GPO Box U1987, Perth, WA 6845, Australia

**Keywords:** Smoke-free policy, Smoking attitudes, University settings, Young people, Theory of Organisational Change

## Abstract

**Background:**

Baseline data were collected to inform the adoption, implementation and institutionalisation phases of a completely smoke free campus policy at a large Western Australian university with a diverse student and staff community.

**Methods:**

An online survey was randomly emailed to staff and students to measure the attitudes towards and the acceptability and enforcement of the policy prior to implementation. In total, 969 respondents completed the survey.

**Results:**

General attitudes towards smoking were negative. While smokers, ex-smokers and non-smokers were supportive of smoke free policy on campus, 65.7% of respondents felt the campus should be completely smoke free. Respondents indicated a smoke free policy should be stringently enforced. The majority of respondents reported that they had been exposed to second-hand smoke on campus (n = 768; 79.5%).

**Conclusion:**

Theory of Organisational Change provides a useful framework to support the implementation of the completely smoke free policy in the University setting. The implementation process needs to consider the broad range of issues associated with implementing a completely smoke free policy and address issues such as safety of smokers, ensuring smokers are not marginalised and ensuring a comprehensive program is implemented. These baseline findings can be used to advocate for the implementation of a comprehensive range of strategies that recognise the addictive nature of tobacco smoking and address attitude and behaviour change, environmental adaptations and effective implementation of the policy. Administration should consider smokers and non-smokers when policy is implemented.

## Background

Tobacco smoking is the most preventable health issue in Australia [1] and worldwide [2]. The negative health consequences of tobacco smoking and exposure to second-hand tobacco smoke are well documented [1].

Australia has a long and successful history of tobacco control interventions. Strategies to address cigarette smoking prevalence have been implemented since the 1970s. Restrictions in federal government workplaces (1986) and on Australian airlines (1987) were implemented, with restrictions in other workplaces, public places and restaurants phased in during the 1980s, 1990s and 2000s [3]. The culture of “exiled smokers” congregating outside office entrances has almost stopped and complete bans now exist at many sports and other outdoor venues [4,5]. These strategies are recognised globally as fundamental to reducing smoking prevalence and associated health risks [1].

Attitudes towards smoking in Australia have changed considerably over the last three decades, mirroring the steadily declining prevalence of smoking in the country [4]. Smoking has become a minority behaviour at a population level with 17.4% of Australians aged 18 years or older reporting smoking on a daily basis in 2010 [1]. However, smoking prevalence continues to be higher among Indigenous Australians (37.6%) and lower socio-economic groups (SES) (24.6%) [1].

Smoking restrictions in the workplace are now common in many jurisdictions with evidence suggesting that smoke free legislation has a positive impact on those who are occupationally exposed [6,7]. Smoke free policies have the potential to improve health outcomes through the elimination of exposure to second-hand smoke. Additionally such policies have the potential to decrease the number of cigarettes smoked and increase cessation among smokers [7], while workplace bans have the capacity to reduce smoking prevalence and daily smoking [8]. Despite debate (usually fuelled by the tobacco industry) that smoking bans would not be accepted by the public; would be difficult to enforce; and would impact on revenue, particularly in the hospitality industry: research has found the contrary [7]. On 1 January 2012 Curtin University became a completely smoke free campus. The policy now restricts smoking in all buildings and vehicles on campus in addition to all grounds and student residential accommodation and grounds. This move to adopt best practice in public health tobacco control provides an opportunity to demonstrate the University’s commitment to the health of its students and staff. However, successful implementation of a smoke free policy at a large University presents a number of challenges including the diversity within the Curtin community with a population that vary in terms of age, SES, education level and cultural background. The large, disparate population of the main campus comprises general and academic staff (approx. n = 5500), approximately 32,000 undergraduate and postgraduate students and significant numbers of visitors. Staff are represented by a variety of occupations and have varying levels of attendance at the main campus depending on employment type.

Curtin University is committed to increasing the combined number of lower SES and Indigenous enrolments to 20% (currently 11%). In Australia low SES groups are those that fall below the third tercile according the Socio-Economic Index for Areas (SEIFA) measures [9]. Australian universities calculate student SES based on home postcode and government student support eligibility [10]. Both low SES and Australian Indigenous groups report higher smoking prevalence compared with the broader community [1]. Further, Curtin has the third largest international student population in Australia with approximately 7,590 studying at the main campus with a significant proportion from low to middle income countries, many of which experience significantly higher smoking prevalence in comparison to Australia [11].

Recent research conducted at Curtin found Australian and New Zealand students were significantly less likely to be current smokers than on-campus international students (9.0% versus 16.9% p < 0.001). In addition, the research reported that male international students were 2.13 times more likely to be a current smoker than domestic students [12]. Many international students reside in the on-site student accommodation which is also completely smoke free.

The demographics and structure of the Curtin University campus population provides a unique opportunity to evaluate the impact of a smoke free workplace initiative in a challenging and diverse setting. However, the addictive nature of tobacco, along with the perceived social and cultural benefits, [4] highlight that appropriate consideration for those who are regular smokers is required. Attitudes of the population need to be assessed to ensure there is appropriate corporate risk management. The physical size of the main campus (116 hectares including large spaces of sports grounds and gardens) and the need for some staff and students to attend campus in the evenings may pose safety concerns. Other studies have found staff are likely to leave the premise to smoke when total bans are implemented [13].

Theory of Organisational Change provides a framework to explain how change in a university setting can be managed. Goodman and colleagues (2002) suggest health promotion interventions in organisations can be managed through four stages including: awareness raising; adoption; implementation and institutionalisation. Raising awareness ensures senior administrators are aware of the health issue. Adoption refers to planning for and adopting policy; implementation focuses on program delivery and capacity building; and institutionalisation is the long-term maintenance of the policy and supporting strategies. Smoking control measures are well established in Australia [14], which according to the Diffusion of Innovation Theory [15] make it an ideal time to implement further restrictions. Diffusion of Innovation theory suggests that behaviour change is initiated by some groups early and this change filters on to other groups. Once a critical mass has changed behaviour social norms begin to change. These changes at a societal level have been evident for smoking behaviour in Australia over the past decades. Changes in norms towards smoking allow for further smoking restrictions to be accepted by the majority of the population.

This research aims to inform the adoption, implementation and institutionalisation phases of a smoke free policy at a large university. The paper describes baseline findings regarding attitudes towards the policy among staff and students and acceptability and enforcement of this policy prior to implementation.

## Methods

### Setting

A random cross sectional sample of staff (n = 500) and students (n = 4500) from all University faculties and facilities were recruited via direct email. Emails were generated by the University Surveys Office, to ensure anonymity. The inclusion criteria for this study were staff and students over the age of 18, enrolled internally and attending the main Curtin campus.

Participants were invited to complete an electronic survey during October 2011. Students were sent two follow-up emails (at one and two weeks after the initial invitation to participate) while staff were sent one follow-up email (two weeks after the initial invitation to participate). This research received ethics approval from the Curtin University Human Research Ethics Committee.

### Survey instrument

The survey instrument was developed using questions from previously validated instruments. These included questions on tobacco use [12], attitudes towards smoking [16] and demographic data [12]. The survey instrument was tested for content validity using an expert panel drawn from health promotion, research and tobacco control (n = 8). Reliability was measured using a test-retest (n = 32) [17]. Changes were made to items with low internal consistency.

#### Smoking status

Tobacco use data were collected using previously validated questions used for students from this University [12]. Respondents were categorised non-smokers if they had never smoked cigarettes or never smoked regularly; ex-smokers if they previously smoked regularly (one cigarette or more per day); and smokers if they currently smoke regularly (more than one cigarette per day) and occasionally (on average, less than one cigarette per day). Respondents were asked how often they had been exposed to second-hand cigarette smoke on campus during the past four weeks.

#### Attitudes towards smoking and smoking restrictions

Attitudes towards smoking items were adapted from previously validated items developed for students from this University [18]. Attitudes towards smoke free restrictions at the University were adapted from a study of Australian TAFE staff [19] (TAFE’s (Technical and Further Education)provide a wide variety of vocational education and training). To measure attitudes towards smoking and attitudes towards smoking restrictions respondents indicated their level of agreement on a five-point Likert scale. Categories were collapsed to agree, neutral and disagree.

#### Awareness of and attitudes towards campus smoking policy

To measure awareness of current campus smoking policy respondents were asked if they were aware of any Curtin policy restricting smoking on campus and asked to indicate ‘yes’, ‘no, or ‘don’t know/not sure’. A subsequent question asked how respondents would describe the university’s current tobacco smoking policy. Attitudes towards the impact of a completely smoke free policy were measured using four items including staff and student quality of life; student learning; and student enrolment using a five-point Likert-type scale. Categories were collapsed to negative, neither negative or positive and positive [20]. A list of six penalties that could apply if individuals do not adhere to smoke free campus policy measured attitudes towards policy enforcement.

### Data analysis

Data were analysed using SPSS for Windows version 20.0. Descriptive analysis of the data provides an overview of participant characteristics. Chi-square analysis was used to determine the impact of a completely smoke free campus for smokers, ex-smokers and non-smokers.

## Results

Of the 5000 staff members and students invited to participate, just under 1000 completed the survey (n = 969; 19.4%). A total of 3,964 (79.3%) did not respond and 67 (1.3%) provided an incomplete response. Demographics of participants are described in Table 1.

**Table 1:** Sample demographics

		**N**	**%**
Sex	Male	362	37.4
	Female	607	62.6
	**TOTAL**	**969**	**100.0**
Primary role	Curtin undergraduate student	599	61.8
	Curtin postgraduate student	141	14.6
	Curtin General/Professional staff member	132	13.6
	Curtin Academic staff member	97	10.0
	**TOTAL**	**969**	**100.0**
International student status	Yes	157	21.3
	No	580	78.7
	**TOTAL**	**737**	**100**

### Second-hand smoke

The majority of respondents reported that they had been exposed to second-hand smoke on campus (n = 768; 80.1%). Approximately one quarter of respondents (23.9%) reported exposure ‘at least daily’ or ‘more than once daily’, 23.5% were exposed a ‘few times a week’, 11.9% ‘once a week’ and 20.8% a ‘few times a month’. Level of exposure was similar for smokers, ex-smokers and non-smokers (p = 0.95). The majority of respondents agreed with the statement ‘*if someone smokes cigarettes around me they are causing me harm because of second-hand smoke’* (84.1%), although there was a significant difference (p < 0.001) between smokers, ex-smokers and non-smokers, with only 46.7% of smokers agreeing with this statement (see Table 2).

**Table 2:** Agreement with tobacco smoking attitude statements reported by University staff and students

	**Non-smokers N (%)**	**Ex-smokers N (%)**	**Smokers N (%)**	**Total N (%)**
If someone smokes cigarettes around me they are causing me harm because of second-hand smoke	686 (89)	86 (80.4)	42 (46.7)	814* (84.1)
I prefer to socialise in a smoke-free environment	695 (90.1)	86 (80.4)	30 (33.3)	811* (83.8)
I seek out smoke-free environments	572 (74.2)	67 (62.6)	13 (14.4)	652* (67.4)
It disappoints me when a friend who normally doesn’t smoke, smokes cigarettes while drinking	547 (70.9)	46 (43)	13 (14.4)	606* (62.6)
I would rather date a non-smoker	701 (90.9)	84 (78.5)	36 (40)	821* (84.8)
I ask others not to smoke around me	354 (44.7)	28 (26.2)	2 (2.2)	375* (38.7)

Agreement with the statement indicated by strongly agree or agree.

*P = <0.001.

### Attitudes towards smoking and smoking restrictions

The general attitudes towards smoking behaviours were negative with most respondents agreeing they ‘*prefer to socialise in a smoke free environment*‘ (83.8%); ‘*would rather date a non-smoker’* (84.8%);‘*seek out smoke free environments’* (67.4%); and were ‘d*isappointed when a friend, who doesn’t normally smoke, smokes while drinking alcohol’* (62.6%). There was a significant difference between smokers, ex-smokers and non-smokers (p < 0.001) with smokers being less likely to agree with these statements compared to ex-smokers and non-smokers. Non-smokers (44.7%) and ex-smokers (26.2%) were more likely to ask others not to smoke around them compared to smokers (2.2% (p < 0.001) (see Table 2).

Both smokers and non-smokers supported a smoke-free policy, although there was some concern about the whole campus being smoke-free. The majority of respondents agreed with the statement that the ‘*Curtin campus should be smoke free in all buildings*’ (n = 884; 91.3%). However, respondents were less likely to agree with the statements ‘*our campus should be completely smoke free*’ (n = 636; 65.7%) and ‘*our campus should be smoke-free including all outdoor areas*’ (n = 589; 60.8%). There was a significant difference (p < 0.001) between smokers, ex-smokers and non-smokers for all responses, with smokers being more likely to disagree with these statements compared to non-smokers and ex-smokers. Over half of respondents (n = 519; 53.6%) agreed that there should be some places on campus where people could go to smoke (see Table 3).

**Table 3:** Agreement with tobacco control attitude statements reported by University staff and students

	**Non-smokers N (%)**	**Ex-smokers N (%)**	**Smokers N (%)**	**Total N (%)**
Our campus should be smoke-free including all out door areas	517 (67.1)	54 (50.5)	18 (20.0)	589* (60.8)
The restrictions on where you can smoke makes it hard for smokers at Curtin University	275 (35.7)	54 (50.5)	44 (48.9)	373** (38.5)
There should be some places at Curtin University where people can go to smoke	377 (48.9)	72 (67.3)	70 (77.8)	519* (53.6)
There should be more help or support at Curtin University for people who want to quit smoking	470 (61)	59 (55.1)	43 (47.8)	572** (59.1)
Because of their professional role, Curtin University staff have a responsibility to be non-smokers	280 (36.3)	20 (18.7)	8 (8.9)	308* (31.8)
Our campus should be smoke-free in all buildings	724 (93.9)	97 (90.7)	63 (70)	884* (91.3)
Our campus should be completely smoke-free	553 (71.7)	63 (58.9)	20 (22.2)	636* (65.7)

Agreement with the statement indicated by strongly agree or agree.

*p < 0.001.

**p < 0.05.

### Awareness of and attitudes towards current campus smoking policy

Over half of the respondents (n = 543; 56%) were aware that the University ‘*had a tobacco policy’*, however, 22.7% (n = 220) were ‘*not aware of the policy’* and 21.3% (n = 206) were ‘*unsure*’. Smokers were more likely to be aware of the existence of a policy (72.2%) compared to ex-smokers (67.3%) and non-smokers (52.5%) (p < 0.001). When this was explored further, 5.2% indicated that there was ‘*a no tobacco policy on campus’*; 9% responded that ‘st*aff, students and visitors are allowed to smoke tobacco in designated areas’* of campus buildings; 58.7% responded ‘*staff, students and visitors are allowed to smoke tobacco in designated areas of the campus grounds but not inside the buildings*’; and 8% responded staff, *‘students and visitors are banned from smoking tobacco throughout the campus; including all campus buildings, grounds and vehicles*’. Almost one-fifth (19.2%) of respondents did not know if there was a policy. There was no significant difference between the responses of smokers, ex-smokers and non-smokers (p = 0.437).

### Attitudes towards a completely smoke free university

The majority of respondents reported that a completely smoke free campus would have a positive effect on staff (70.4%) and student (74.7%) quality of life. Just over half of respondents (56%) suggested a smoke free campus would have a positive effect on student learning and 40.8% suggested it would have a positive effect on student enrolments. There was a significant difference (p < 0.001) between smokers, ex-smokers and non-smokers for all items with non-smokers reporting that the policy would have a more positive impact on student learning and student enrolments than ex-smokers and non-smokers (see Table 4).

**Table 4:** Effect of a completely smoke free campus policy

	**Non-smokers N (%)**	**Ex-smokers N (%)**	**Smokers N (%)**	**Total N (%)**	**P value**
**Staff quality of life**					<0.001
Negative	40 (5.2)	9 (8.4)	20 (22.2)	69 (7.1)	
Neither negative or positive	149 (19.3)	27 (25.2)	42 (46.7)	218 (22.5)	
Positive	582 (75.5)	71 (66.4)	28 (31.1)	681 (70.4)	
**Student quality of life**					<0.001
Negative	40 (5.2)	8 (7.5)	21 (23.3)	69 (7.1)	
Neither negative or positive	112 (14.5)	29 (27.1)	35 (38.9)	176 (18.2)	
Positive	619 (80.3)	70 (65.4)	34 (37.8)	723 (74.7)	
**Student learning**					<0.001
Negative	31 (4.0)	6 (5.6)	18 (20)	55 (7.9)	
Neither negative or positive	260 (33.7)	59 (55.1)	52 (57.8)	371 (38.3)	
Positive	480 (62.3)	42 (39.3)	20 (22.2)	542 (56)	
**Student enrolment**					<0.001
Negative	63 (8.2)	17 (15.9)	15 (16.7)	95 (9.8)	
Neither negative or positive	360 (46.7)	58 (54.2)	60 (66.7)	478 (49.4)	
Positive	348 (45.1)	32 (29.9)	15 (16.7)	395 (40.8)	

Respondents indicated enforcement of a smoke free policy on campus should include: reminders (n = 319; 32.9%); a monetary fine (n = 200; 20.9%); disciplinary process for staff/students (n = 237; 24.5%); community service (n = 200; 20.6%); and anti-smoking education (n = 319; 32.9%); while 5.6% indicated there should be no consequences (n = 54) for individuals not adhering to the policy.

## Discussion

This study provides insight into the smoking prevalence, attitudes and behaviours of staff and students at a large Australian university. The findings indicate that the prevalence of any smoking in this study was low (9.8%), compared to smoking rates among the Australian adult population (17.4%) [1]. However, the prevalence is similar to data previously collected from 17–24 year old students at this University (9.8% current daily smoking) [12] and from Australian TAFE staff (8.1% current daily smoking; 1.8% occasional smoker) [19]. The low prevalence of smoking may however be due to selective non-response and under-reporting, with another university study suggesting non-respondents are more likely to be smokers than respondents [21].

Males were slightly underrepresented comprising only 37.7% of study participants. In 2011, 43% of staff members were male. Similarly, males comprised 46.4% of the 2011 student population. International students were under represented (21.3%) however they comprise 39.7% of the student population. Another study at this University also found males and international students to be underrepresented in online surveys [12].

Attitudes towards cigarette smoking were generally negative which reflects general attitudes in Australia [22]. Similar to Western Australian trends [23] it was common for respondents of this study to prefer to socialise in smoke free environments (83.8%) and to date a non-smoker (84.8%). However respondents were less likely to ask others not to smoke around them (38.7%).This is consistent with other research which has found that ‘unempowered’ non-smokers in particular are not likely to act on smoking by others [24] which suggests a need to motivate and provide necessary skills to enable individuals and the community to vocalise concerns around exposure to secondary smoke.

Theory of Organisational Change highlights the importance of the adoption and implementation phase for tobacco control [25]. Less than half of respondents (44%) were aware of the University’s current tobacco policy restrictions. School based studies have reported awareness of the policy and appropriate enforcement are essential to ensure compliance [26-28]. The implementation phase should include awareness raising strategies to ensure staff, students and visitors are aware of the new policy so as to encourage high levels of support. Additionally, reinforcing messages highlighting the adverse health effects of second-hand smoke have been found to improve acceptance and compliance with smoke free policies [29]. Understanding the social and organisational norms that might support or undermine a smoking ban, will lead to more successful implementation of the policy.

There is a dearth of published data describing the impact of smoke-free policy on Australian Higher Education Campuses. Similar to studies on college campuses in the USA [30,31] respondents in this study generally supported a smoke free policy, however, were less supportive of a complete ban on all campus grounds. While the majority of respondents in this study (91.3%) were supportive of campus buildings being smoke free, respondents were less likely to suggest the campus should be completely smoke free (65.7%). Given the acceptance of policy implementation and the positive changes in social norms towards smoking over the last few decades in Australia the reluctance from these respondents may reflect a conflict between norms of distal groups and the norms held by an individual or an individual’s proximal reference groups [32,33]. These attitudes may also be influenced by feelings of concern around stigma smokers may experience [34]. In their study Poland and colleagues (2012) found ‘reluctant’ and ‘easygoing’ smokers were supportive of smoking restrictions as long as they were implemented sensitively and supported with appropriate messages. Australian data has found smoking restrictions in public places (11.2%) and at workplaces (7%) to be motivators to successfully quit smoking [1]. In view of these findings it is anticipated a complete smoking ban is also likely to positively contribute to social norms of this population group. Most respondents in this study reported exposure to second-hand smoke (79.5%) and most agreed that second-hand smoke was harmful to their health (84.1%). There is a body of research that describes the effectiveness of a smoke free policy in reducing the harm associated with exposure to second-hand smoke [7,35]. Increasing the awareness of the health benefits that a total campus ban on smoking provides is essential when implementing a smoke free policy [29]. Health has been found to be the main motivator for quitting smoking; followed by the cost [1]. Consistent with these findings the implementation phase of the policy [25] should incorporate motivators for change; in particular an emphasis on the health and economic benefits of quitting smoking.

Despite the proven benefits in terms of reducing exposure to second-hand smoke [7,35] and the successes in reducing the prevalence of cigarette smoking [6,22] there are health issues that need to be considered during the adoption and implementation of a workplace smoke-free policy. Research indicates that workplace smoke-free bans (WSB) have a positive relationship with self-perceived work-related stress, especially among males and young adults (18–40 years) [36]. Additionally, it has been reported that employees who left workstations to smoke outside their building, smoked their cigarettes 19% ‘harder’ than in social settings [37].

Due to addictive nature of nicotine, smokers may seek private areas to smoke within a restricted workplace. This may have implications as staff experience isolation, guilt and stigma. Others have found smoke free workplaces to contribute to stigma among smokers [34] and there is a need for evaluation of the unintended negative effects of policy for smokers [38]. The safety of smokers is also an issue for staff and students on a large university campus, as they may seek unsafe private areas to smoke in, moving from offices or classrooms to the outskirts of the campus including in the evenings. It may also impact negatively on those students living in on-campus housing who are no longer able to smoke in the grounds of their ‘home’. Studies have reported negative consequences, especially in regard to safety issues for women, who smoke outside bars [39]. These issues pose challenges for implementation and significant efforts should be made to ensure the needs of smokers are addressed. Consistent with best practice in health promotion [40] a comprehensive range of strategies, which include support for smokers, are most likely to have optimal outcomes.

Enforcement of a smoke-free policy is imperative and has been found to be predictive of successful implementation. School based research suggests consistently enforced policy is the best predictor of adherence [26-28]. Respondents in this study suggested a range of transparent enforcement strategies. Although almost three-quarters of respondents (students: 74.7%; staff: 70.4%) reported that a total smoke-free policy would have a positive impact on staff and student quality of life, the reported positive impact on student learning (56%) and student enrolments(40.8%) were much less. Given that the attitudes towards a smoke-free campus are positive, the association with a better quality of life is implicit and may indicate a better acceptance of the policy.

### Limitations

Although the response rate to this study was low, the results provide a snapshot of attitudes towards policy implementation at a large and diverse university campus. Respondents were slightly biased in that a greater proportion of non-smokers, females and domestic students participated compared to the university demographics however others have also found this to occur in university studies [21]. Selective non-response and underreporting may have biased these results. The disproportionate representation of non-smokers may have resulted in more positive attitudes towards the policy than smokers may have presented. As is consistent with previous data from this university [12] the prevalence of smoking was lower than the general Australian population. Other studies have found non-responders are more likely to be smokers [21]. The limitations of the study should be considered when reviewing the results.

## Conclusions

Smoke-free policies can reduce harms associated with second-hand smoke [35] and have the potential to reduce the number of cigarettes smoked and increase smoking cessation [7,41]. As a proportion of staff and students support smoke free areas, but not a completely smoke free campus, the adoption and implementation phases should include strategies to raise the awareness of the importance of policy in reducing harms associated with second-hand smoke and in encouraging cessation. Involving smokers in the planning process is likely to ensure strategies are relevant. The physical size and the use of the campus in the evening highlight the need for appropriate strategies to ensure the safety of smokers. Appropriate enforcement of the policy is necessary for adherence. Despite the proven benefits of smoke free policy administrators should acknowledge issues a totally smoke free policy may pose, especially for smokers.

## Competing interests

The authors declare that they have no competing interests.

## Authors’ contributions

SB and JJ have made substantial contributions to the conception and design. All authors reviewed the article for important intellectual content. NB and SB made substantial contributions to data analysis. All authors read and approved the final manuscript.

## Pre-publication history

The pre-publication history for this paper can be accessed here:

http://www.biomedcentral.com/1471-2458/13/738/prepub

## References

[B1] Australian Institute of Health and Welfare 2010 National Drug Strategy Household Survey report. Drug statistics series no. 25. Cat. no. PHE 145 2011 Canberra: Australian Institute of Health and Welfare

[B2] World Health Organization WHO report on the global tobacco epidemic 2011: Warning about the dangers of tobacco 2011 Geneva: Organization World Health Organization

[B3] White V Hill D Siahpush M Bobevski I How has the prevalence of cigarette smoking changed among Australian adults? Trends in smoking prevalence between 1980 and 2001 Tob Control 2003 12 Suppl ll ii67 ii74 12878776 10.1136/tc.12.suppl_2.ii67 PMC1766106

[B4] Chapman S Freeman B Markers of the denormalisation of smoking and the tobacco industry Tob Control 2009 17 25 31 18218803 10.1136/tc.2007.021386

[B5] Walsh RA Paul CL Paras L Stacey F Tzelepis F Workplace-related smoking in New South Wales: extent of bans, public attitudes and relationships with relapse Health Promot J Aust 2011 22 2 85 90 10.1071/he11085 21819348

[B6] Goodman PG Haw S Kabir Z Clancy L Are there health benefits associated with comprehensive smoke-free laws Int J Public Health 2009 54 367 378 10.1007/s00038-009-0089-8 19882106

[B7] Hyland A Barnoya J Corral JE Smoke-free air policies: past, present and future Tob Control 2012 21 2 154 161 10.1136/tobaccocontrol-2011-050389 22345239

[B8] Fichtenberg CM Glantz S Effect of smoke-free workpalces on smoking bahviour: a systematic review Br Med J 2002 325 7357 188 10.1136/bmj.325.7357.188 12142305 PMC117445

[B9] Statistics ABo Socio-economic indexes for areas (SEIFA) 2011 2013 Canberra: Australian Bureau of Statistics

[B10] Bradley D Noonan P Nugent H Scales B Review of Australian Higher Education: Final Report 2008 Canberra: Commonwealth of Australia

[B11] World Health Organization WHO report on the global tobacco epidemic, 2009: implementing smoke-free environments 2009 France: World Health Organisation

[B12] Howat P Hallett J Kypri K Maycock B Dhaliwal S McManus A Tobacco smoking in an Australian university sample and implications for health promotion Prev Med 2010 51 425 426 10.1016/j.ypmed.2010.08.015 20817020

[B13] Watson D Glover M McCool J Bullen C Adams B Min S Impact of national smokefree environments laws on teachers, schools and early childhood centres Health Promot J Aust 2011 22 3 166 171 10.1071/he11166 22497058

[B14] Wood L Sullivan D Donovan R Australia TCCoW Public education campaigns on smoking in Western Australia: their evolution and effects The progress of tobacco control in Western Australia: achievements, challenges and hopes for the future 2008 Perth: Cancer Council of Western Australia

[B15] Rogers EM Diffusion of preventive interventions Addict Behav 2002 27 989 993 10.1016/S0306-4603(02)00300-3 12369480

[B16] Lee L Dhaliwal S Howat P University student attitudes to smoking policies 2008 Perth, Western Australia: Western Australian Centre for Health Promotion Research and Centre for Behavioural Research in Cancer Control

[B17] Windsor R Baranowski T Clark N GC Evaluation of health promotion, health education and disease prevention programs 1994 Mountain View: Mayfield Publishing Company

[B18] Le L Dhaliwal S Howat P University student attitudes to smoking policies 2008 Curtin University: Perth Western Australian Centre for Health Promotion Research and Centre for Behavioural Research in Cancer Control

[B19] Bonevski B Paul CL Walsh RA Support for smoke-free vocational education settings: an exploratory survey of staff behaviours, experiences and attitudes Health Promot J Aust 2011 22 1 11 16 10.1071/he11011 21717831

[B20] Berg CJ Lessard L Parelkar PP Thrasher J Kegler MC Escoffery C Goldade K Ahluwalia JS College student reactions to smoking bans in public, on campus and at home Health Educ Res 2011 26 1 106 118 10.1093/her/cyq076 21123843 PMC6433431

[B21] Kypri K Baxter J Smoking in a New Zealand university sample New Zeal Med J 2004 117 1 6 15107897

[B22] Chapman S Australia CCW Introduction The progress of tobacco control in Western Australia: achievements, challenges and hopes for the future 2008 Perth: Cancer Council Western Australia

[B23] Carter O Australia CCW Changes in the attitudes and beliefs of Western Australian smokers, 1984–2007 The progress of tobacco control in Western Australia: achievements, challenges and hopes for the future 2008 Perth: Cancer Council Western Australia

[B24] Poland BD Cohen JE Ashley MJ Adlaf E Ferrence R Pederson LL Bull SB Raphael D Heterogeneity among smokers and non-smokers in attitudes and behaviour regarding smoking and smoking restrictions Tob Control 2000 9 364 371 10.1136/tc.9.4.364 11106705 PMC1748400

[B25] Goodman RM Steckler A Kegler MC Glanz K, Rimmer BK, Viswanath K Mobilizing organisations for health enhancement: theories of organisational change Health Behavior and Health Education 2002 San Francisco, CA: Jossey-Bass

[B26] Evans-Whipp TJ Bond L Ukoumunne OC Toumbourou JW Catalano RF The impact of school tobacco policies on student smoking in Washington State, United States and Victoria, Australia Int J Environ Res Public Health 2010 7 698 710 10.3390/ijerph7030698 20616998 PMC2872326

[B27] Sabiston CM Chris Y Lovato CY Ahmed R Pullman AW Hadd V Campbell HS Nykiforuk C Brown S School smoking policy characteristics and individual perceptions of the school tobacco context: are they linked to students’ smoking status? Journal Youth Adolescence 2009 38 1374 1387 10.1007/s10964-009-9422-z PMC2758151 19779813

[B28] Øverland S Aarø LE Lindbak RL Associations between schools’ tobacco restrictions and adolescents’ use of tobacco Health Educ Rev 2010 25 5 748 756 10.1093/her/cyq023 20363827

[B29] Borland R Yong H Siahpush M Hyland A Campbell S Hastings G Cummings K Fong G Support for and reported compliance with smoke-free restaurants and bars by smokers in four countries: findings from the International Tobacco Control (ITC) Four Country Survey Tob Control 2006 15 iii34 iii41 10.1136/tc.2004.008748 16754945 PMC2593054

[B30] Loukas A Garcia MR Gottlieb NH Texas college students’ opinions of no-smoking policies, secondhand smoke, and smoking in public places J Am Coll Health 2006 55 27 32 10.3200/JACH.55.1.27-32 16889312

[B31] Rigotti NA Regan S Moran SE Wechsler H Students’ opinion of tobacco control policies recommended for US colleges: a national survey Tob Control 2003 12 25 26 10.1136/tc.12.suppl_1.i25 PMC1747763 12958381

[B32] Lewis MA Neighbors C Gender-specific misperceptions of college student drinking norms Psychol Addict Behav 2004 18 334 339 15631605 10.1037/0893-164X.18.4.334

[B33] Liefbroer AC Billari FC Bringing norms back in: a theoretical and empirical discussion of their importance for understanding demographic behaviour Popul Space and Place 2010 16 287 305

[B34] Stuber J Galea S Link BG Smoking and the emergence of a stigmatized social status Soc Sci Med 2008 67 3 420 430 10.1016/j.socscimed.2008.03.010 18486291 PMC4006698

[B35] Centers for Disease Control and Prevention Reduced secondhand smoke exposure after implementation of a comprehensive statewide smoking Ban–New York, June 26, 2003–June 30, 2004 Morb Mortal Wkly Rep 2007 56 705 708 17637596

[B36] Azagba S Sharaf MF The association between workplace smoking bans and self-perceived, work related stress among smoking workers BMC Publ Health 2012 12 123 10.1186/1471-2458-12-123 PMC3296657 22329920

[B37] Chapman S Haddad S Sindhusake D Do work-place smoking bans cause smokers to smoke “harder”? Results from a naturalistic observational study Addiction 1997 95 5 607 610 9219383

[B38] Houle B Siegel M Smoker-free workplace policies: developing a model of public health consequences of workplace policies barring employment to smokers Tob Control 2009 18 64 69 10.1136/tc.2008.026229 19168490 PMC2722043

[B39] Moore R Annechino R Lee J Unintended consequences of smoke-free bar policies for low-SES women in three California countries Am J Prev Med 2009 37 2 S138 S143 10.1016/j.amepre.2009.05.003 19591753 PMC2730500

[B40] Howat P Maycock B Cross D Collins J Jackson L Burns S James R Towards a more unified version of health promotion Health Promot J Aust 2003 14 2 82 85

[B41] Kouvonen A Kivimäki M Oksanen T Pentti J Heponiemi T Väänänen A Virtanen M Vahtera J Implementation of workplace-based smoking cessation support activities and smoking cessation among employees: the finnish public sector study Am J Public Health 2012 102 7 e56 e62 10.2105/AJPH.2012.300823 22594722 PMC3477988

